# Subjectivity study on health conservation of elderly hemodialysis patients

**DOI:** 10.1186/s12877-024-04819-3

**Published:** 2024-02-27

**Authors:** Eunji Yim, Mijin Yun, Sohyune Sok

**Affiliations:** 1 Department of Nursing, Hyejeon College, Chungcheongnam-do, Republic of Korea; 2 Department of Nursing, Graduate School, Kyung Hee University, Seoul, Republic of Korea; 3 College of Nursing Science, Kyung Hee University, 26, Kyungheedae-Ro, Dongdaemun-Gu, Seoul, 02447 Republic of Korea

**Keywords:** Aged, Health conservation, Hemodialysis, Subjectivity, Q methodology

## Abstract

**Background:**

Health conservation enables elderly hemodialysis patients to maintain a positive state of well-being while undergoing treatment and maintenance of disease. This study was to identify the type of perceptions on health conservation of elderly hemodialysis patients and compare the characteristics of perceptions.

**Methods:**

This study used an exploratory study design applying Q methodology, which is designed to research subjectivity. The study determined a population of subjective statements, the concourse, based on the preceding literature and interviews with twenty-five elderly patients over 65 years of age with hemodialysis. We chose a total of 50 statements considered to be representative of the concourse for the Q-sample. The study selected 50 elderly patients over 65 years of age with hemodialysis as the P-set. The participants provided their internal viewpoints by sorting the Q-sample items into a grid. The researchers performed an analysis using PC-QUANL program. Data were collected from June to November, 2019.

**Results:**

Type I, ‘support system-based effort’ focused on one’s own effort, positive and proactive attitude, family support, medical instructions, information, and medications. Type II, ‘skeptical life maintaining’ expressed a pessimistic future without hope, strongly negative perception on preserving health, and thus minimal effort and motivation to continue life. Type III, ‘treatment process interest’ is based on an interest in the hemodialysis process; for them, it is important to follow medical staff’s instructions, take regular medications precisely, pay attention to the results of regular monthly blood tests, and control their health. Type IV, ‘positive effort’ accepts hemodialysis positively, lives with hemodialysis, and carries out all daily life activities.

**Conclusion:**

In nursing practice, nurses need to pay attention to the perceptions on health conservation of elderly hemodialysis patients. This study can be implied as the evidence of nursing practice based on the perception on health conservation of elderly hemodialysis patients.

## Introduction

Among the hemodialysis patients, the proportion of elderly patients aged 65 years or older is rapidly increasing from 33.8% in 2009 to 63.7% in 2020 [1]. Along with the aging process, elderly hemodialysis patients experience physical, psychological, and social problems. In addition to physical problems, such as fatigue, dry skin, sleep disturbance, itching, and bone disease [2–5], they have psychological problems such as depression and anxiety [2, 4]. They also have social problems due to economic burden and absence of a spouse [4]. A nursing approach to the health management of elderly hemodialysis patients is required due to the necessity of health management for disease-related complications, regular dialysis and medication, diet control, arteriovenous fistula, exercise and rest, blood pressure, and weight [2, 5, 6].

The health awareness of elderly hemodialysis patients is heavily dependent on medical staff and dialysis machines, with the treatment focusing on dialysis and medication, and they strongly believe that the disease itself must be treated chronically [5, 7]. Since they relied on medical staff and dialysis machines for so long, elderly hemodialysis patients had a lower sense of control over their diseases than that of patients with diabetes or asthma [4, 5]. In the study result of Shim [8], there were 1.4 times more patients who thought their health had deteriorated compared to the previous year than those who thought theirs had improved. Patients often found it difficult to even come for hemodialysis treatment, which is essential for prolonging their lives, and complained of despair and a hopeless future for having to undergo dialysis until death [3]. For elderly hemodialysis patients who have a low sense of control over their disease, health condition worsening every year, and the pain of despair and hopeless future, it is impossible to recover to their pre-disease state. Therefore, a new solution is necessary in order to preserve their current health from deteriorating. According to Sung [9], nursing for the elderly should focus on maintaining or preserving the functions of the elderly rather than healing so that their health status does not deteriorate. Conservation should be a prerequisite to health promotion, and health through conservation should be well maintained in order to maintain the health of elderly hemodialysis patients [9, 10].

Conservation refers to “keeping together” or “maintaining the proper balance” [11]. It is a source that helps us in effectively coping with difficult problems that arise during the aging process [12]. In addition, it is the interaction of the four domains: personal integrity, social integrity, structural integrity, and conservation of energy [11]. This applies to an individual responding and adapting to the crisis in his or her own way, and continuing to function as a whole in a crisis situation [11]. Based on this, health conservation is a compound word that combines the concepts of health and conservation. It is defined as “maintaining a state of physical, mental, and social well-being” or “maintaining a balance as a physical, mental, social, and psychological unity” [9].

In the study on health conservation, Choi [13] focused on the effects of family support and self-efficacy on health conservation in elderly hemodialysis patients. There have been no studies on people’s various attitudes, values, beliefs, and subjectivity toward health conservation. Many other health conservation studies investigated the relationship between variables and health conservation targeting the elderly and middle-aged women [3–6, 9, 10].

Health conservation enables the elderly to maintain a positive state of well-being while undergoing treatment and maintenance of disease [6, 10]. In the case of the elderly with chronic diseases, it is important to preserve their functions so that they can perform daily activities independently rather than simply extending their lifespan or taking a treatment-oriented approach [9]. Therefore, elderly hemodialysis patients should focus on developing a plan for maintaining a positive state of well-being by managing their disease through health conservation and maintaining their current health.

Health conservation is determined by their values, beliefs, and attitudes rather than variables such as family support and self-efficacy [3, 4]. As a result, the existing quantitative research makes it difficult to explore the health conservation of elderly hemodialysis patients, who have their own health conservation method [3, 14]. Elderly hemodialysis patients need to preserve their health in their own way based on their life experiences and inclinations [5, 14]. In the case of the elderly, their psychological and spiritual health may act as a motive for maintaining and preserving their health compared to the other age groups [5, 15]. In order to explore the individual subjective understanding of health conservation in elderly hemodialysis patients, it is necessary to apply Q-methodology, an approach to human psychological characteristics that ‘understands from the inside’ rather than ‘explains from the outside’ [16]. Q-methodology is useful for uncovering subjective opinions or cognitive structures. Q-methodology can uncover subjective attitudes, beliefs, and values in depth because it starts from the actor’s point of view instead of an external observer, the researcher [5, 16, 17]. Therefore, Q-methodology can be considered as a suitable method for exploring the health conservation of the elderly hemodialysis patients in this study.

The purpose of this study was to apply Q-methodology to explore the types of subjective perceptions on the health conservation of elderly hemodialysis patients, analyze the structure of each type of subjective perception on the health conservation of elderly hemodialysis patients, and provide fundamental data for nursing intervention strategies.

## Methods

### Study design

This study used an exploratory study design applying Q-methodology, which is a subjective research method, to explore the types of subjective perception on the health conservation of elderly hemodialysis patients.

### Selection of concourse

In-depth interviews on health conservation with elderly hemodialysis patients were conducted for 22 days (August 23, 2019 to September 13, 2019). The study included 25 elderly patients aged 65 years or older who had been receiving hemodialysis for at least 6 months at two hemodialysis clinics in Seoul. In-depth interviews on the health conservation of elderly hemodialysis patients were conducted with questions centered on the four attributes of conservation by Levine [11]. During the interview process, questions were asked naturally, and additional questions were asked based on the interview content. Following the interview, transcriptions were completed as soon as possible based on the participants’ recordings, and the non-verbal communication observed during the interview were recorded in parentheses. Field notes were prepared, which included the atmosphere of the field, impressions received by the researcher, and observations by the researcher. The in-depth interview was conducted by repeatedly listening to and reading the interview data organized in this way, taking notes of missing parts and considerations for the next interview, and supplementing it in the next interview.

### Extracting and organizing Q-sample

The Q-sample was selected from the concourse, the sum of all opinions, and statements related to health conservation during the in-depth interview process. Statements related to health conservation were extracted while repeatedly listening to and reading the content recorded during the interview process of the elderly hemodialysis patients. The number of first extracted statements was 170, which were categorized and classified by sub-attributes in the four areas of conservation. Duplicate items were deleted, ambiguous items were corrected, and 60 statements were extracted a second time.

During the extraction and classification of the Q-statements [18], the statements were refined with the help of 1 professor of nursing, 1 professor of Q-methodology, 2 persons with Ph.D. in nursing, 2 doctoral students of nursing with Q-research experience, 2 nephrologists currently treating elderly hemodialysis patients, and 2 chief nurses who have been working in the hemodialysis room for more than 20 years. Finally, opinions were exchanged during the review process of one elderly hemodialysis patient on whether the statements clearly reveal the subjectivity of the participants. A Q-sample of the final 50 items was extracted, with statements that clearly represented the subdimensions of each attribute of conservation.

### Selection of Q-sample

Q-sample refers to the items extracted from the concourse. It was selected based on the attributes of conservation by Levine [11], so that irrelevant or unnecessary statements were not included. The final Q-sample selected through the Q-statement extraction and arrangement process included 50 items. The Q-sample size may vary depending on the unique nature of the study, with 40 to 60 items being common [16, 19]. Given the significance of achieving a balance of positive, negative, and neutral items, the Q-sample was adjusted in order to have an equal number of positive and negative items (18 positive items, 18 negative items, and 14 neutral items) [16, 20]. The Q-sample of 50 items selected for health conservation in elderly hemodialysis patients consisted of the attributes of conservation by Levine [11], including 14 items for personal integrity, 8 items for social integrity, 15 items for structural integrity, and 13 items for conservation of energy.

### Q-sort

In regard to the Q-sort, the time requirement was explained to the P-set of 50 persons, and data were collected. Since the subjects were elderly patients with chronic diseases, the sorting was conducted in the lounge within the hemodialysis room, where they could request help from the medical staff in case of fatigue or emergency during sorting.

The researcher provided and read instructions to the participants prior to the Q-sort process and created an environment where they could talk and sort in the most comfortable atmosphere possible. During the Q-sort process, the Q-cards were shuffled and the statements were read one after the other. They were classified into three categories: agree (positive), neutral (indifferent, no feeling), and disagree (negative) according to the importance of one’s subjective opinion. The sorted cards were classified on a 9-point scale from the item most agreed with to the item most disagreed with according to the importance of one’s subjective opinion.

Following the Q-sort, participants were asked about their placement of the statements at both extreme ends of the sorting grid. The time required for Q-sort, filling out the questionnaire, and interview ranged from 45 to 90 min, an average of 60 min.

### Ethical consideration

This study was approved by Institutional Review Board of a University in Seoul, South Korea (IRB No. KHSIRB-18086(RA)). All information related to the research design, methods, and procedure effects were explained sufficiently so that the study participants could understand them. IRB-approved explanations were provided to all study participants, and they were informed that they could withdraw from the study at any time, and that there would be no disadvantages afterwards, and strict confidentiality was ensured. In order to safely protect the participants’ personal information, the questionnaire was collected immediately after filling out on site, and all information related to the study was stored in a double-locked personal locker. This study was conducted from June to November, 2019.

### Data analysis

To extract the factors, PCA was used, and Varimax rotation was used. The interpretation and naming of the final Q-factors were based on the factor arrays such as the standard score of each statement as well as the characteristics of the participants and the contents of the interview. The naming of the types was discussed with one professor with expertise in Q-methodology, one professor of geriatric nursing, one professor of journalism, two medical professors treating elderly patients on hemodialysis, and one elderly patient who had been on hemodialysis for 8 years.

## Results

### Types of health conservation

Types of health conservation in elderly hemodialysis patients are shown in Tables 1, 2 and 3. Health conservation in elderly hemodialysis patients was classified into 4 factors and, therefore, 4 types. Items that showed agreement regardless of type among all Q-items are referred to as consensus items. In regard to the items with a ‘positive’ consensus among types, 1 item with an average standard score of +1.00 or higher was ‘I want to maintain my current health because promoting health has its limits’, and 2 items with an average standard score of lower than -1.00 were ‘I think I can be managed with drugs even if I do not go on a diet’ and ‘I do not follow the hospital’s instructions, and I eat whatever I want’ (Table 3).

**Table 1:** Eigen value and variance by factor

	Factor I (Type I)	Factor II (Type II)	Factor III (Type III)	Factor IV (Type IV)
Eigen value	14.00	3.07	2.28	2.20
Variance (%)	28.00	6.15	4.57	4.41
Cumulative (%)	28.00	34.15	38.72	43.13

**Table 2:** Correlations by the types of health conservation

	Type I	Type II	Type III	Type IV
Type I	1.00	0.36	0.58	0.58
Type II		1.00	0.32	0.31
Type III			1.00	0.63
Type IV				1.00

**Table 3:** Consensus Q-items among types

Q-items	Mean standard score
15. I want to maintain my current health because there is a limit to promoting health.	1.29
9. Dialysis-related support (emotional and physical support) by the medical staff helps to conserve health.	0.88
14. I am adjusting well to my changed daily life with hemodialysis.	0.45
3. I am receiving hemodialysis because I will die otherwise.	0.36
5. Enduring 4 h of hemodialysis is the most difficult.	-0.20
30. I get a lot of stress during hemodialysis.	-0.63
32. I have no religion or belief, and I only believe in myself.	-0.69
18. I do not follow the hospital’s instructions, and I eat whatever I want.	-1.15
22. I think I can be managed with drugs even if I do not go on a diet.	-1.44

### Characteristics by type

The participant distribution on the factors was 15 for Type I, 6 for Type II, 14 for Type III, and 12 for Type IV. The subjective perceptions on health conservation in elderly hemodialysis patients were named ‘support system-based effort type’, ‘skeptical life maintaining type’, ‘treatment process interest type’, and ‘positive effort type’ considering the characteristics of each type.

#### Type I: ‘support system-based effort’

The Q-items that showed strong agreement in Type I consisted of 9 statements in the order: ‘The most important thing for health conservation is one’s own efforts’ (Z score = 1.97), ‘Family is the most helpful when it comes to health conservation’ (Z score = 1.78), ‘For health conservation, I try to follow the instructions of the medical staff at the hemodialysis hospital’ (Z score = 1.75), ‘I must take medicines as directed by the hospital’ (Z score = 1.55), ‘I have to be strong for health conservation’ (Z score = 1.54), ‘A positive and active mindset is beneficial to health conservation’ (Z score = 1.41), ‘I want to maintain my current health because there is a limit to promoting health’ (Z score = 1.34), ‘I accept dialysis as my fate’ (Z score = 1.11), and ‘The information related to hemodialysis provided by the medical staff is helpful for health conservation’ (Z score = 1.02).

The items that showed relatively strong disagreement in Type I were 8 statements in the order: ‘I am in despair because I have no hope for the future’ (Z score = -1.96), ‘I find the mental symptoms associated with hemodialysis to be extremely difficult’ (Z score = -1.81), ‘My fellow patients contribute to health conservation’ (Z score = -1.65), ‘I have wanted to die numerous times ever since I started hemodialysis’ (Z score = -1.57), ‘Going to a meeting rather than staying at home is helpful for health conservation’ (Z score = -1.49), ‘I think I can be managed with drugs even if I do not go on a diet’ (Z score = -1.31), ‘Religion is a huge help in my hemodialysis life’ (Z score = -1.29), and ‘The physical symptoms related to hemodialysis are too difficult for me’ (Z score = -1.28).

#### Type II: ‘skeptical life maintaining’

The Q-items that showed strong agreement in Type II consisted of 9 statements in the order: ‘I must take medicines as directed by the hospital’ (Z score = 1.99), ‘The physical symptoms related to hemodialysis are too difficult for me’ (Z score = 1.68), ‘I need to exercise regularly for health conservation’ (Z score = 1.68), ‘I want to maintain my current health because there is a limit to promoting health’ (Z score = 1.52), ‘I make an effort each day, but there is no hope for the future’ (Z score = 1.40), ‘For health conservation, I try to follow the instructions of the medical staff at the hemodialysis hospital’ (Z score = 1.15), ‘Government subsidy for hemodialysis costs is helpful for health conservation’ (Z score = 1.15), ‘My appearance has become shabby as I had hemodialysis’ (Z score = 1.13), and ‘We need national and local welfare policies for the elderly who are living alone and receiving hemodialysis’ (Z score = 1.05).

The items that showed relatively strong disagreement in Type II were 9 statements in the order: ‘Going to a meeting rather than staying at home is helpful for health conservation’ (Z score = -1.90), ‘Family is the most helpful when it comes to health conservation’ (Z score = -1.78), ‘I think I can be managed with drugs even if I do not go on a diet’ (Z score = -1.72), ‘My fellow patients contribute to health conservation’ (Z score = -1.56), ‘I have my own knowledge of health conservation’ (Z score = -1.54), ‘Religion is a huge help in my hemodialysis life’ (Z score = -1.32), ‘I couldn’t admit that I was sick at the beginning of hemodialysis, so I couldn’t go on a diet’ (Z score = -1.11), ‘I am gaining a better understanding of hemodialysis through education’ (Z score = -1.02), and ‘I do not manage blood vessels separately’ (Z score = -1.00).

#### Type III: ‘treatment process interest’

The Q-items that showed strong agreement in Type III consisted of 9 statements in the order: ‘For health conservation, I try to follow the instructions of the medical staff at the hemodialysis hospital’ (Z score = 2.11), ‘I must take medicines as directed by the hospital’ (Z score = 1.64), ‘A positive and active mindset is beneficial to health conservation’ (Z score = 1.48), ‘I pay attention to the blood test result performed every month and strive to conserve my health’ (Z score = 1.44), ‘I want to maintain my current health because there is a limit to promoting health’ (Z score = 1.27), ‘I try to restrict my water and salt intake at every meal’ (Z score = 1.22), ‘The most important thing for health conservation is one’s own efforts’ (Z score = 1.20), ‘I take adequate rest (after dialysis, after exercise, and in daily life)’ (Z score = 1.11), and ‘During hemodialysis, I actively communicate with the medical staff for necessary requirements (blood pressure measurement, warmth, thirst, and body weight)’ (Z score = 1.07).

The items that showed relatively strong disagreement in Type III were 11 statements in the order: ‘I have wanted to die numerous times ever since I started hemodialysis’ (Z score = -1.96), ‘I am in despair because I have no hope for the future’ (Z score = -1.90), ‘My fellow patients contribute to health conservation’ (Z score = -1.62), ‘I make an effort every day, but there is no hope for the future’ (Z score = -1.59), ‘I am not worried about my health because the medical staff provide good hemodialysis management’ (Z score = -1.40), ‘I do not follow the hospital’s instructions, and I eat whatever I want’ (Z score = -1.26), ‘I have no religion or belief, and I only believe in myself’ (Z score = -1.16), ‘I do not manage blood vessels separately’ (Z score = -1.13), ‘I wonder if it’s worth doing this until I die’ (Z score = -1.06), ‘This result was caused by my failure to take care of my health in terms of alcohol intake, tobacco use, and diet prior to starting hemodialysis’ (Z score = -1.04), and ‘I think I can be managed with drugs even if I do not go on a diet’ (Z score = -1.02).

#### Type IV: ‘positive effort’

The Q-items that showed strong agreement in Type IV consisted of 7 statements in the order: ‘The most important thing for health conservation is one’s own efforts’ (Z score = 2.68), ‘I have to be strong for health conservation’ (Z score = 2.12), ‘I need to exercise regularly for health conservation’ (Z score = 2.02), ‘A positive and active mindset is beneficial to health conservation’ (Z score = 1.67), ‘I do my best every day’ (Z score = 1.08), ‘I want to maintain my current health because there is a limit to promoting health’ (Z score = 1.04), and ‘Family is the most helpful when it comes to health conservation’ (Z score = 1.01).

The items that showed relatively strong disagreement in Type IV were 9 statements in the order: ‘I think I can be managed with drugs even if I do not go on a diet’ (Z score = -1.69), ‘I do not follow the hospital’s instructions, and I eat whatever I want’ (Z score = -1.64), ‘When I first thought about receiving hemodialysis, I felt like the sky was falling’ (Z score = -1.63), ‘Receiving dialysis is so hard that I have to lie down for at least two hours at home on the day of dialysis’ (Z score = -1.51), ‘I couldn’t admit that I was sick at the beginning of hemodialysis, so I couldn’t go on a diet’ (Z score = -1.44), ‘This result was caused by my failure to take care of my health in terms of alcohol intake, tobacco use, and diet prior to starting hemodialysis’ (Z score = -1.28), ‘I am not worried about my health because the medical staff provide good hemodialysis management’ (Z score = -1.23), ‘I am well aware that I will have to undergo hemodialysis for the rest of my life, but it is difficult to come and receive hemodialysis’ (Z score = -1.18), and ‘I am in despair because I have no hope for the future’ (Z score = -1.12).

## Discussion

Type I is the ‘support system-based effort type’. It focuses on the personal integrity of one’s effort and positive and active mindset, the social integrity of family help, and the structural integrity of instructions and information from medical personnel and medication. Positive thinking is said to promote ego integrity in the elderly [12, 21], and the more positive the perspective and attitude toward old age, the better chances one has of maintaining their current health status and preparing for life in old age [21, 22]. According to Sung [9], personal integrity had the highest score, and the threat of self-esteem among the elderly made it difficult to maintain their health.

In order to preserve health through amicable relationships and mutual cooperation with Type I patients, families, and medical staff, positive values must first be given in order to promote ego integrity so that efforts for health conservation can be continued with a positive mindset. For Type I ‘support system-based effort type’, a nursing intervention strategy should be prepared by inspiring positive thinking and utilizing the support resources of family and medical staff.

Type II is a ‘skeptical life maintaining type’, with no hope for the future, falling into a sense of hopelessness, suffering from hemodialysis side effects, and sometimes having thoughts of death. As a result, patients of this type exert minimal effort to sustain life. This type ranks second with a variance of 6.15%, and it has a strong negative perception of the future. However, hope improves the well-being, disease-coping ability, and health status of hemodialysis patients [2, 23]. It also encourages active coping skills in the process of achieving goals based on the expectation that good conditions will continue or the condition will improve, and difficult situations will be overcome [2, 12, 24].

Meanwhile, in the Q-methodology research for self-management in hemodialysis patients, the ‘pessimistic reality atrophic type’ had limited interpersonal relationships as they were unable to engage in economic activities due to dialysis, resulting in psychological atrophy and depressed feelings, having a negative mindset, and lacking self-confidence due to the life threats associated with dialysis [5]. In this study, negative emotions caused by life threats in hemodialysis life were similar to negative emotions about the future regarding incurable diseases [5]. Adult patients with end-stage renal disease often experience atrophy in economic activity; however, a difference was found in this study as less than 30% of elderly hemodialysis patients engage in social activities or meetings. According to the life experience study of long-term hemodialysis patients by Park [3], ‘heavy with despair and regret’ and ‘no hope for the future’ were similar to Type II of this study. Steps in converting Type II negative emotions to positive emotions are as follows. First, encourage the patients to express their negative emotions. Second, provide the elderly hemodialysis patients with the support of the national system and family so that they can engage in economic activities or social life as social activities give them positive meaning and value to life and the ability to cope effectively when faced with situations [14, 25]. Third, it is important to develop a program that will promote positive emotions, including hope, for elderly hemodialysis patients [26, 27]. It is necessary to prepare a nursing intervention strategy that will increase their self-esteem and convert negative emotions using these methods.

Type III is the ‘treatment process interest type’. Based on the interest in the hemodialysis process, it is important for patients of this type to follow the instructions of the medical staff, to take their regular medicine properly, to pay attention to the blood test result performed on a monthly basis and follow the diet, and to strive to control their body weight between hemodialysis through a diet with limited water and salt intake, focusing on maintaining health rather than promoting health. This type focuses on structural integrity. Chang [10] emphasized that the focus should be placed on physiological adaptation in old age due to the decline in immunity caused by aging.

In order to successfully perform the hemodialysis treatment process in Type III patients, it is necessary to build a trusting relationship with the medical staff along with the patient’s own efforts. The medical staff should provide the elderly patients with accurate information related to hemodialysis, including blood tests, and educate them on the importance of taking their medications. Furthermore, the medical staff and the patient should agree on a treatment goal in order to prepare a nursing intervention strategy that will help them in achieving their goal. Patients should be involved in complications management, diet control, blood vessel management, and blood pressure management during the treatment process so that they can make their own decisions and participate in the treatment process in order to maintain constant interest.

Type IV is the ‘positive effort type’, which focuses on personal integrity by accepting hemodialysis positively, doing their best in the changed daily life while on hemodialysis, and making efforts for themselves. Positive thinking was a significant predictor of health conservation for the elderly [10, 24]. It is not the external event itself that determines an individual’s happiness and unhappiness in old age, but one’s own belief system about the external event, implying that a positive attitude is the foundation of healthy adaptation [22, 28]. In a subjectivity study on the self-management of hemodialysis patients, the ‘passionate self-directed type’ showed an active effort to overcome the situation at the center of treatment rather than simply worrying about the need for hemodialysis due to a disease, and intended to prevent complications that might occur if self-management was not achieved [5]. Type IV has a higher self-efficacy than the other types, and tries to see the positive side of the disease and treatment process. In order to enhance these strengths, it is necessary to provide a program to promote an active attitude, support the elderly hemodialysis patients so that they can preserve their health on their own, and prepare a nursing intervention strategy that can be implemented.

## Limitations

In this study, there are limitations in not considering demographics, such as age, region, economic status, presence of guardians, and health status, that can affect health conservation in elderly hemodialysis patients. Also, the failure to fully consider individuals’ lives, environments, experiences, culture, and values, such as their thoughts, relationships, and values, which may affect health conservation, may be a limitation of this study. However, the results of Q methodology research, like qualitative research, are about theory development. In Q methodology research, generalization is not statistical inference about the population, but substantive inference that connects generalization about a phenomenon to the population [29]. It is argued that the generalization problem of Q methodology can be examined in terms of the concept of “reliable schematics”. The results obtained from the exploration of two pairs of “tandem-study” strongly demonstrate the schematically reliable nature of Q-study results generated from different investigations of the same subjective phenomenon [30]. Therefore, this study may contribute to construct and present future research and to indicate study direction as a subjective study of qualitative research.

## Conclusions

In conclusion, the four perception types for health conservation in elderly hemodialysis patients were Type I “support system-based effort”, Type II “skeptical life maintaining”, Type III “treatment process interest”, and Type IV “positive effort type”. In the structure of the perception for health conservation in elderly hemodialysis patients based on the analysis of the four types confirmed through the above results, striving for health conservation with positive and active mindset, following the instructions of the medical staff based on family help, taking medications properly, and exercising regularly were meaningful.

Q methodology used this study provides the ability to differentiate views, rather than examining a single aggregate view that might not actually represent any real view of these patients. Based on the results of this study, we can plan to introduce various types of health preservation to prevent deterioration of the current condition for hemodialysis elderly patients whose lives are nearing an end. We can use the research results to practice health preservation efforts without giving up. Especially, screening patients for providing counseling maybe be practiced based on the results of this study to hemodialysis elderly patients. In addition, based on the results of this study, by applying patient care and treatment processes to suit the unique type of each elderly hemodialysis patient, the conservation of the patient’s health can be strengthened and ultimately the quality of their life can be improved.

The results of this study are expected to contribute to health conservation in elderly hemodialysis patients by using type-specific interventions tailored to individual characteristics rather than uniformed patients care.

## Data Availability

The original contributions presented in the study are included in the article, further inquiries can be directed to the corresponding author.
